# Comparison of polymerization behaviors, microhardness and compressive strength between bulk-fill resin and dual-cured core resin

**DOI:** 10.1186/s12903-025-06353-z

**Published:** 2025-07-01

**Authors:** Hye Jeong Kim, Jiyoung Kwon, Hyun-Jung Kim, Reuben H. Kim, Duck-Su Kim, Ji-Hyun Jang

**Affiliations:** 1 Department of Conservative Dentistry, Graduate School, Kyung Hee University, Seoul, Korea; 2 Department of Conservative Dentistry, School of Dentistry, Kyung Hee University, 26 Kyungheedae-ro, Dongdaemun-gu, Seoul, 02447 Korea; 3 Section of Restorative Dentistry, School of Dentistry, University of California Los Angeles, Los Angeles, CA USA

**Keywords:** Bulk-fill resin, Dual-cured core resin, Polymerization shrinkage, Depth of cure, Microhardness, Compressive strength

## Abstract

**Objectives:**

This study aimed to investigate the polymerization shrinkage, microhardness, DOC, and compressive strength of composite resin materials employed for core restoration, encompassing dual-cured core resin composites and LC bulk-fill composites.

**Methods:**

Bulk-fill resin composites [Filtek One Bulk fill (BFO), Bulk Fill Base (BBB), Metafil Bulkfill One (BMF)] and dual-cured core resin composites (Luxacore Z, Any-Core) served as the experimental group, while two conventional LC resin composites [Filtek Z350 (FTZ), metafil flo (FMF)] served as the control group. Two dual-cured core resin composites were named as follows, CLD, CLS (Luxacore Z, in dual-cured mode and self-cured (SC) mode); CAD and CAS (Any-Core, in dual-cured mode and SC mode). Linear polymerization shrinkage, depth of cure (DOC) using microhardness, and compressive strength were evaluated for each experimental group. The results were analyzed using one-way ANOVA, and a post-hoc test (Duncan’s test) was performed at a 95% confidence level.

**Results:**

The linear polymerization shrinkage rate was higher in the dual-pol9ymerization core resin group than in the control group FTZ and bulk-fill resin groups, and in each dual-cured core resin, the dual-cured group was higher than the SC group. The DOC was higher in the bulk-fill resin group than in the control group, and the DOC of the dual-cured core resin group showed a significant variation depending on the product. The compressive strength of the bulk-fill resin group showed a significant variation depending on the product. The compressive strength in the bulk-fill resin group was higher as the filler content increased in the dual-cured mode than the SC mode in the dual-cured core resin group.

**Conclusions:**

Bulk-fill resins showed superior polymerization shrinkage and DOC compared to conventional resin composites and dual-cured core resins. At 4 mm depth, Bulk-fill resin composites achieved an appropriate DOC, but for dual-cured core resins, the DOC varied depending on the product.

**Clinical significance:**

Highly filled bulk-fill resins could be a predictable restorative material for deep cavities and post-endodontic core material, considering its polymerization shrinkage, DOC, and mechanical properties.

## Introduction

Teeth with severe damage to the upper part of the structure from decay, injury, previous unsuccessful treatment, habits such as teeth grinding, or post-endodontic treatment need a core build-up to restore the shape and provide enough support [1, 2]. Various materials like dental amalgam, glass ionomer, resin-modified glass ionomer, resin composite, and ceramic are recommended to rehabilitate the tooth structure. Among these materials, composite resin cores are preferred because they can bond to dental structures and have superior mechanical properties [3, 4].

In terms of core restoration, light-cured (LC) resin composites face challenges caused from their geometrical deep cavities or endodontically treated teeth, which include incomplete solidification in the lower part of the cavity due to limited light penetration compared to the depth of cure (DOC), polymerization shrinkage, color stability, and degree of conversion (DC) [5–8]. These factors could affect the success and longevity of the restoration.

The DOC, which shows how thick a properly solidified resin composite is, is limited by light absorption and scattering within the materials [9]. When a resin composite solidifies, dimethacrylate monomers change from weak forces between molecules to strong bonds, causing solidification shrinkage [10–12]. The mechanical properties of resin composites are directly affected by the DC achieved during solidification. The DC shows the number of double bond carbons in a monomer that change to single bonds during solidification. During this process, aromatic amines change into free radicals.

Dual-cured resin composites were introduced to address the limitations of DC and improve the DOC in deep cavities like post-endodontic restorations [6, 13, 14]. These composites use both chemical and light-induced solidification initiation [1]. When dual-cured resin composite is placed in a deep cavity, solidification mainly happens through photo-initiated chemical reactions at the top and through chemical initiation at the bottom [13]. However, insufficient chemical solidification in the deep parts of the core material, due to insufficient intensity of light solidification or even absence of light solidification, could lead to incomplete solidification [1, 13]. Even though the dual-cured resin composite is designed to start solidifying through two mechanisms, free radical generation by chemical and light solidification, the presence or absence of light, and the quality of light activation significantly affect various properties of the dual-cured resin core, like DOC, solidification shrinkage, and DC [1, 9, 15]. Arrais et al. found that the self-curing (SC) mode gave lower hardness values compared to the light-curing mode, no matter the product [16]. Spinell et al. reported that dual-cured resin composites had a higher DC when light-cured than when relying on self-solidification, and the DC of dual-cured resin was not related to the filler content [17].

To overcome the limitations associated with normal composite resin restoration, like solidification shrinkage and insufficient DOC, the layering filling method has been widely adopted [10]. However, this method needs additional time in the dental chair for filling and light solidification [18, 19]. Also, it increases the risk of trapping air bubbles or moisture between each layer of resin composites, which can cause recurrent caries related to increased exposure to the environment of the biodegradable matrix metalloproteinases level [11].

The bulk-fill resin composites were introduced to make the restoration process more efficient and minimize chair time allowing bulk solidification in one application [18, 20]. According to the manufacturers, bulk-fill resin composite maintains a good degree of solidification and depth even at 4 mm due to improved filler and shows mechanical properties similar to those of regular resin composites [21, 22]. Bulk-fill LC composite materials have been reported to offer several potential benefits in dental restorative procedures, including increased efficiency, reduced risk of air pockets and contamination, and minimized solidification shrinkage [10, 23]. The aim of this study was to investigate the solidification shrinkage, microhardess, DOC, and compressive strength of composite resin materials used for core restoration, including both dual-cured core resin composites and LC bulk-fill composites.

## Materials and methods

### Materials

Bulk-fill resin composites and dual-cured core resin composites served as the experimental group, while two conventional LC resin composites served as the control group. The sample size was determined based on preliminary data, with a significance level (α) of 0.05 and a desired statistical power of 0.80. Specifically, for the compressive strength and linear polymerization shrinkage tests, twelve specimens per group (*n* = 12) were used. For the depth of cure and microhardness tests, five specimens per group (*n* = 5) were used, and measurements were performed three times per specimen.

A high-viscosity bulk-fill (Filtek One Bulk fill (BFO), 3 M EPSE, St Paul MN, USA) and two low-viscosity bulk-fill resin composites (Bulk Fill Base (BBB), Sun Medical, Moriyama, Japan; Metafil Bulkfill One (BMF), Sun Medical) were used as bulk-fill experimental groups. Two dual-cured core resin composites were used for core resin composites in two different modes, which were the dual-cured mode and the SC mode, with or without light-polymerization, respectively. Those groups were named as follows, CLD, CLS (Luxacore Z, DMG, Hamburg, Germany, in dual-cured mode and SC mode); CAD and CAS (Any-Core MEDICLUS, Cheongju, Korea, in dual-cured mode and SC mode).

Two conventional LC resin composites, a nanofilled composite (Filtek Z350 (FTZ), 3 M EPSE) and microfilled flowable resin (metafil flo (FMF), Sun Medical), were used as the control groups.

The chemical compositions and materials used in this study are listed in Table 1.

**Table 1:** Chemical composition of experimental materials used in this study

Group	Material	Manufacturer (Lot No)	Chemical composition	Filler content (wt%/vol%)
*Conventional hybrid flowable composite (control)*				
FMF	Metafil Flo (A2)	Sun Medical, Moriyama, Japan (FS2)	Barium silica glass, colloidal silica *Filler particle size: 0.01–10 μm* UDMA	65/44
*Conventional Nanofilled composite (control)*				
FTZ	Filtek Z350 XT (A2)	3 M EPSE, St.Paul MN, USA (9703694)	Silica, zirconia, aggregated zirconia/silica cluster filler *Filler particle size: 4–20 nm* Bis-GMA, UDMA, TEGDMA, Bis-EMA, PEGDMA	78.5/63.3
*Bulk-fill Low-viscosity composite resin*				
BBB	Bulk Base	Sun Medical, Moriyama, Japan (VM12)	Barium glass filler, strontium aluminosilicate glass *Filler size: 1–5 μm* Bis-MPEPP, urethane acrylate	70/46
BMF	Metafil Bulk Fill One	Sun Medical, Moriyama, Japan	silane treated Barium silica glass *Filler size: 0.02–2 μm* UDMA, EBPADMA	74/54.3
*Bulk-fill High-viscosity resin composite resin*				
BFO	Filtek One Bulk fill	3 M EPSE, St.Paul MN, USA (NF14711)	Silica, zirconia, ytterbium trifluoride agglomerates *Filler particle size: 4–20 nm* UDMA, 1,12-DDMA, AFM, AUDMA	76.5/58.4
*Dual-cure core composite resin*				
CLD^**^ CLS^**^	LuxaCore Z (white)	DMG, Hamburg, Germany (272057)	Zirconium oxide, barium glass, pyrogenic silicic acid *Filler size: 0.01–4 μm* Bis-GMA	72/50
CAD^**^ CAS^**^	Any-Core (A3)	Mediclus, Cheongju, Korea (AC38T897)	Barium glass, silicone dioxide *Filler size: undisclosed* Bis-GMA, TMPTMA, EDMAB	60/67

*Abbreviations: *UDMA* urethane dimethacrylate, *Bis-GMA* bisphenol A diglycidildimethacrylate, *TEGDMA* triethylene glycol dimethacrylate, *Bis-EMA* Ethoxylatedbisphenol A dimethacrylate, *PEGDMA* polyethylene glycol dimethacrylate, *bis-MPEPP* Bisphenol A polyethoxy methacrylate, *EBPADMA* Ethoxylated bisphenol A dimethacrylate, *1*,*12-DDMA* 1, 12-dodecanediol dimethacrylate, *AFM* additional fragment monomer, *AUDMA* aromatic urethane dimethacrylate, *TMPTMA* Trimethylolpropane trimethacylate, *EDMAB* ethyl-4-dimethylaminobenzoate

** *CLD* LuxaCore Z with light curing, *CLS* LuxaCore Z without light curing, *CAD* Any-Core with light curing, *CAS* Any-Core without light curing

### Measurement of the linear polymerization shrinkage

To examine the polymerization shrinkage, twelve specimens were tested for each group using µ-Biomechanics (IB systems, Seoul, Korea). Composite samples with a diameter of 6 mm and with 0.5 mm thickness of prepared.

For the light-polymerization groups, which included control groups, LC bulk-fill groups and dual-cured core in dual-cured mode groups, the specimens were positioned beneath the tip of a linear variable differential transformer (LVDT) probe and the linear polymerization shrinkage values were measured for 700 s. After preparation, baseline measurements were taken without light exposure for the first 10 s, followed by light-polymerization for 20 s (SmartLite Focus, Dentsply Sirona, Milford, DE, USA).

For the SC mode of the dual-cured core resin composite, the specimens were positioned beneath the tip of the LVDT probe, and the linear polymerization shrinkage values were measured for 1800 s without light exposure.

The polymerization shrinkage rate was calculated by the below equation. $\:\varDelta\:h$ represented final shrinkage amount at 700s (µm) and $\:h$ represented thickness of composite after polymerization (µm).

$$\:Shrinkage\:rate\:\left(\%\right)=\frac{\varDelta\:h}{\varDelta\:h\:+\:h}\times\:100\:\left(\%\right).$$

### Measurement of the depth of cure and Vickers microhardness

To examine DOC in the experimental groups, Vickers microhardness tests were performed. Five specimens from each group were prepared in a cylindrical shape with 4 mm diameter x 4 mm height by using a polyacrylic mold (Dentsply Caulk, Dentsply Sirona, Milford, DE, USA).

For the light-polymerization groups, which included the control groups, LC bulk-fill groups, and core resin composites in the dual-cured mode groups, the top surface of the specimen was light-cured for 30 s (SmartLite Focus, Dentsply Sirona), followed by 24 h of storage in the dark at room temperature. The mold did not allow light transmission from other directions, and a polyester strip was positioned over the top and bottom surfaces to standardize the surface roughness.

For the SC mode of core resin composite, the resin composites were placed in polyacrylic mold (Dentsply Caulk, Dentsply Sirona), followed by 24 h storage in dark space at room temperature. Polyester strips were positioned over the top and bottom surfaces to standardize the surface roughness.

The Vickers microhardness was determined on the top and the bottom surfaces of each specimen using an HMV-G31ST (Shimadzu, Kyoto, Japan). The bottom/top microhardness ratio (B/T ratio), which is the microhardness ratio between the upper and lower surfaces, was calculated to measure DOC. The Vickers microhardness was measured three times for each specimen. For each reading, a 0.5 kgf (4.903 N) load was applied over the specimen’s surface for 5 s. The results were evaluated using a microhardness tester stereomicroscope at 10× magnification. The reading microhardness value (HV), which is a dimensionless unit defined as the applied load divided by the surface area of the indentation, was determined by automatic calculations.

The microhardness values of the upper surfaces were compared to determine the physical properties of each group, as the microhardness values of the core material surface were crucial.

### Measurement of compressive strength

Twelve specimens of each group were prepared in a cylindrical shape with 4 mm diameter x 6 mm height using a polyacrylic mold to examine the compressive strength [24, 25].

For the light-polymerization groups, which included control groups, LC bulk-fill groups and dual-cured cores in the dual-cured mode groups, the specimens were cured for 20 s (BluePhase20i, Ivoclar Vivadent, Schaan, Liechtenstein) and stored in the dark for 24 h at room temperature.

The dual-cured core in the SC mode group, resin composites were not light-activated and polymerized in the dark for 24 h at room temperature. Polyester strips were positioned on the top and bottom surfaces to standardize the surface roughness.

The compressive strength test was performed using a universal testing machine (Autograph AG-IS; Shimadzu, Kyoto, Japan) at a crosshead speed 0.5 mm/min.

### Statistical analysis

The data were analyzed using one-way ANOVA followed by Duncan’s post hoc comparison at a significance level of 0.05. All statistical analyses were performed using SPSS version 25.0 (IBM SPSS Inc, Armonk, NY, USA). For both polymerization shrinkage, microhardness tests and compressive strength, the sample size was determined based on data of preliminary test using the significance level of 0.05 and desired power of 0.80.

## Results

### Linear polymerization shrinkage

**Fig. 1:**
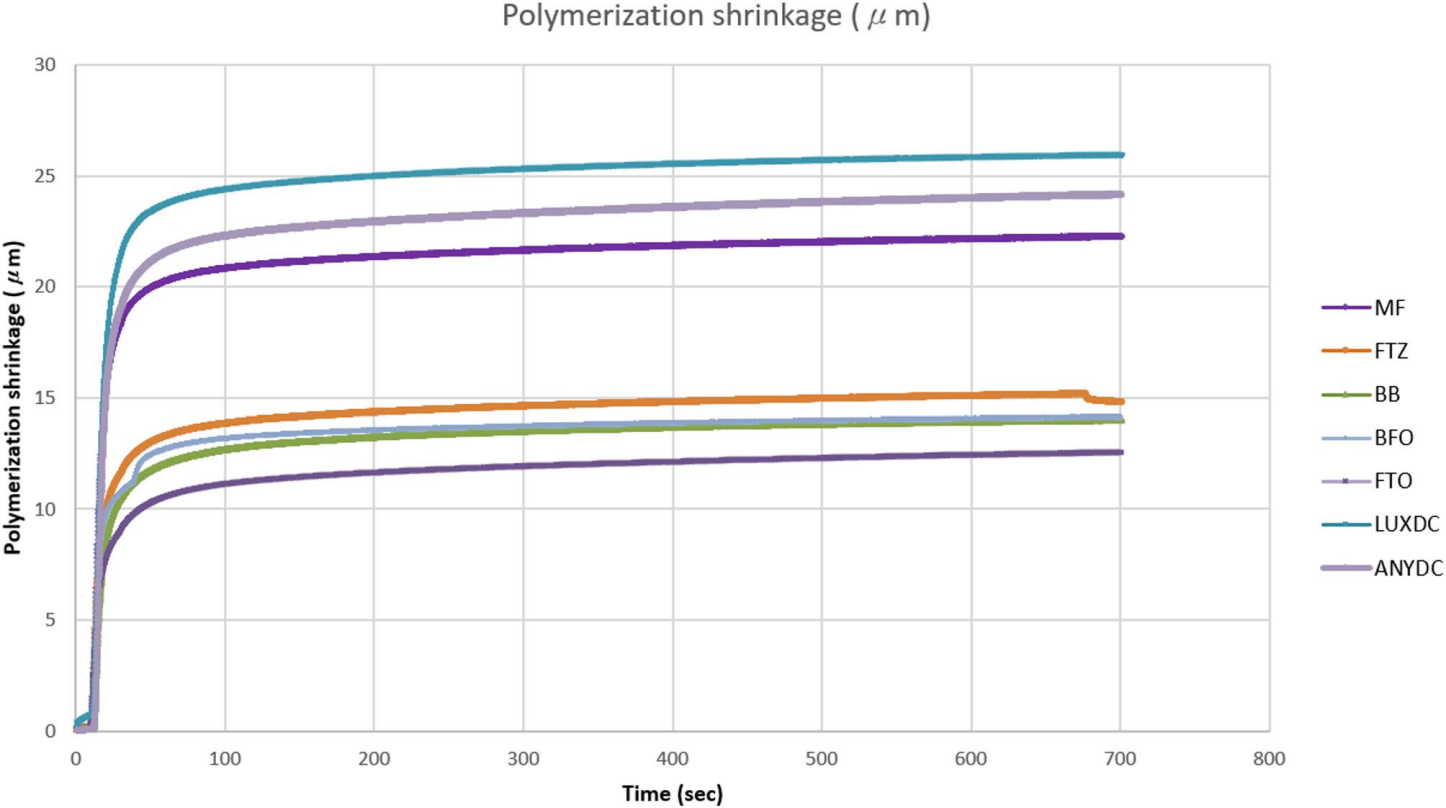
Linear polymerization shrinkage over 700 s after light curing

The amount of linear polymerization shrinkage varies depending on the material used. However, the overall patterns of linear polymerization shrinkage for the light-polymerization groups showed a steep polymerization shrinkage during 20 s of light curing and a gradual shrinkage up to 700 s (Fig. 1).

**Fig. 2:**
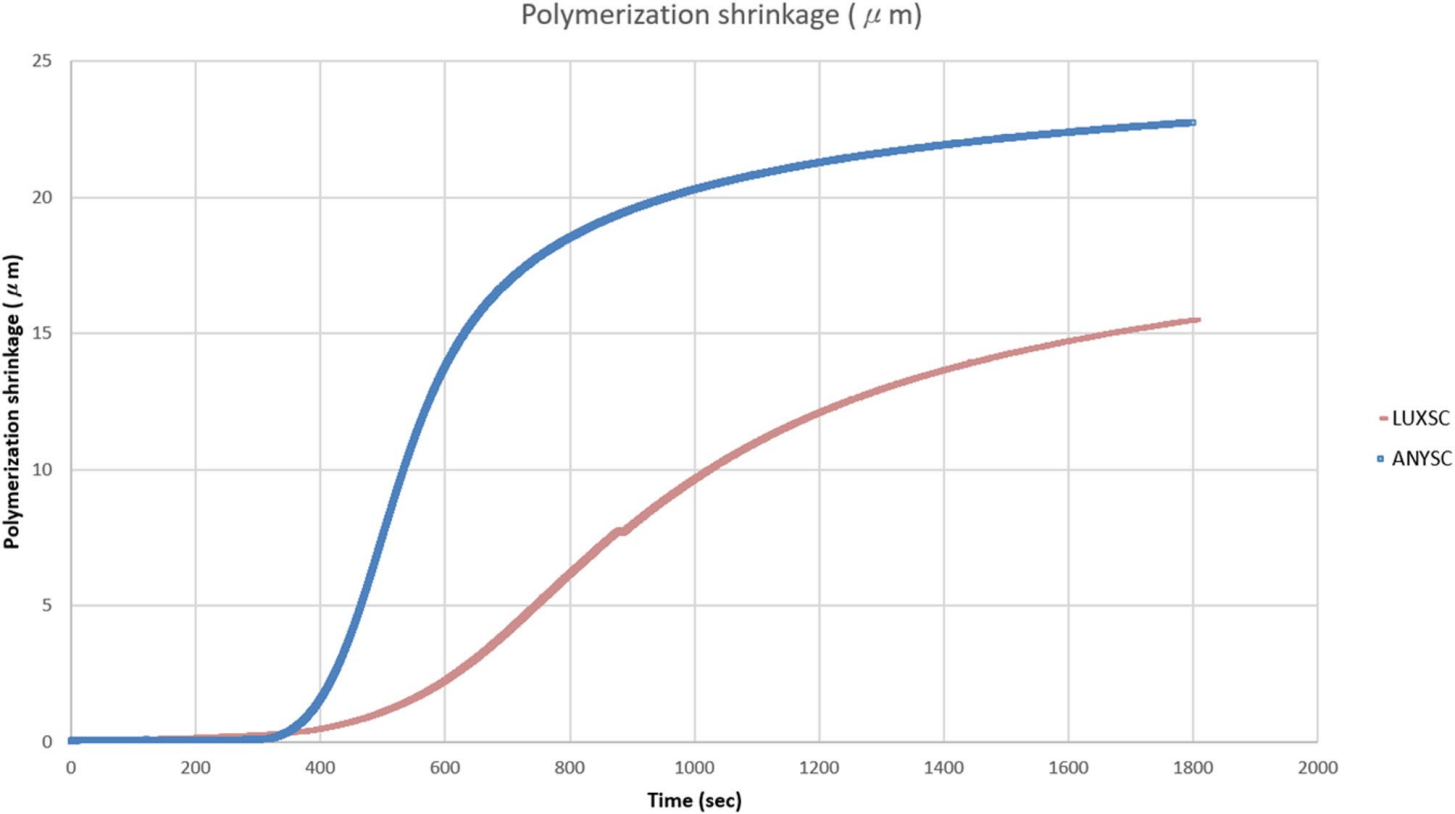
Linear polymerization shrinkage over 1800 s without light curing

In the SC groups of the core resin composites (Fig. 2), the linear polymerization shrinkage exhibited almost deficient levels of contraction in the initial 200–300 s, but then showed a gradually increasing pattern over 1800 s.

**Fig. 3:**
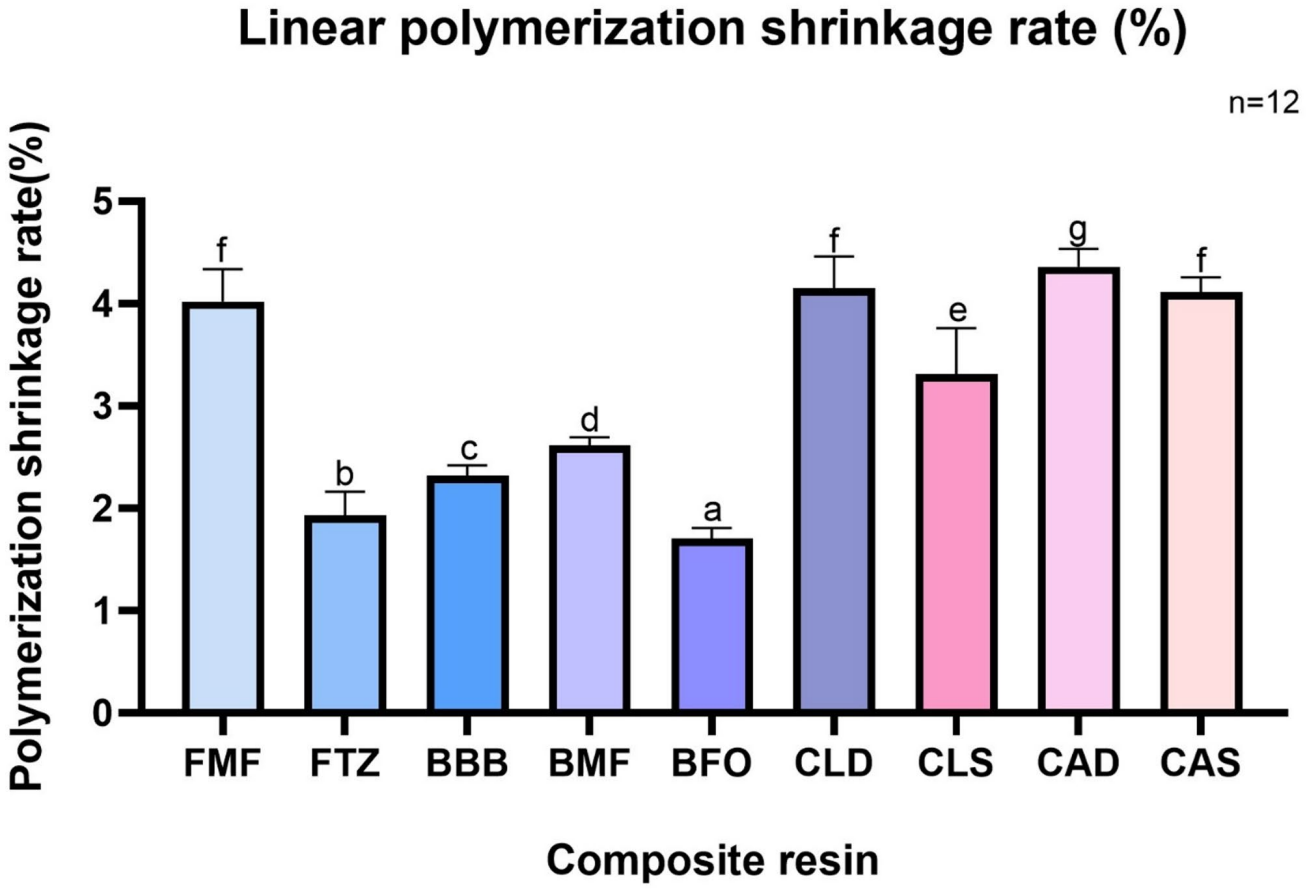
**Linear polymerization shrinkage rate (%).** Different lowercase letters represent statistically significant differences (*p* < 0.05)

The results of the linear polymerization shrinkage and shrinkage rate of the experimental groups measured at the endpoint are shown in Fig. 3; Table 2. The linear polymerization shrinkage rate varied depending on the resin composite material. The order increased in the following order: BFO < FTZ < BBB < BMF < CLS < CLD ≈ FMF, CAS < CAD (*p* < 0.05).

**Table 2:** **Linear polymerization shrinkage amount (µm) and rate (%) at the endpoint***n* = 12

Groups	Polymerization shrinkage rate (%)
FMF	4.019 (0.30) ^f^
FTZ	1.931 (0.22) ^b^
BBB	2.316 (0.10) ^c^
BMF	2.757 (0.08) ^d^
BFO	1.708 (0.10) ^a^
CLD	4.154 (0.30) ^f^
CLS	3.311 (0.43) ^e^
CAD	4.360 (0.17) ^g^
CAS	4.116 (0.13) ^f^

*n* = 12, Data are presented as mean (SD)

Mean values with different superscript lowercase letters represent statistically significant differences (*p* < 0.05)

BFO, a high-viscosity nanofilled bulk-fill resin composite, showed the lowest linear polymerization shrinkage rate among all experimental groups. FTZ, a nanofilled conventional resin composite, exhibited a higher shrinkage rate than BFO.

Among the flowable low-viscosity resins, BBB and BMF, which are bulk-fill resin composites, exhibit significantly lower linear polymerization shrinkage rates than FMF, a conventional flowable resin. BBB showed significantly lower shrinkage than BMF. The order of polymerization shrinkage among the bulk-fill resins was BFO < BBB < BMF (*p* < 0.05).

Among the dual-cured resin core groups with or without light-curing, CAD exhibited the highest linear polymerization shrinkage rate, followed by CAS, CLD, and CLS.

The bulk-fill resin groups showed a significantly lower linear polymerization shrinkage rate than the dual-cured resin core groups, regardless of light-activation (*p* < 0.05).

### Depth of cure

B/T ratio values were shown in Table 3. BMF and CAS showed the highest B/T ratios among the experimental groups, whereas FMF showed the lowest B/T ratio.

**Table 3:** Vickers microhardness (HV) and bottom/top microhardness ratio (%)

Group	Top	Bottom		B/T Ratio (%)
FMF	33.56 (4.85) ^b^	9.61 (2.08) ^a^	28.61 (4.22) ^a^	
FTZ	76.29 (4.81) ^f^	47.65 (4.63) ^f^	62.67 (7.06) ^c^	
BBB	23.04 (1.61) ^a^	19.21 (1.06) ^b^	83.74 (7.17) ^e^	
BMF	43.93 (3.18) ^c^	39.77 (2.47) ^e^	90.61 (1.88) ^f^	
BFO	58.82 (3.37) ^e^	46.25 (3.82) ^f^	78.54 (2.79) ^de^	
CLD	50.89 (5.13) ^d^	19.68 (9.64) ^b^	39.72 (19.91) ^b^	
CLS	Unmeasurable	Unmeasurable	Unmeasurable	
CAD	35.36 (1.17) ^b^	26.58 (2.32) ^c^	75.06 (4.57) ^d^	
CAS	34.86 (2.19) ^b^	31.57 (1.66) ^d^	90.85 (6.43) ^f^	

Data are presented as mean (SD)

Mean values with different superscript lowercase letters represent statistically significant differences (*p* < 0.05)

DOC was considered adequate when the mean values corresponded to a value equal to or higher than 80% of the surface readings [26]. CAS, BMF and BBB exhibited over 80% B/T ratio in the whole experimental groups, which were 90.85 (6.43), 90.61 (1.88), and 83.74 (7.71), respectively. BFO fell short of the B/T ratio of 80% but was close to it.

Among the bulk-fill resin composites, BMF exhibited the highest B/T ratio, followed by BBB and BFO. All of these showed a higher B/T ratio than the control resin groups, FMF and FTZ.

Among the dual-cured core resin groups, CLS was not sufficiently polymerized to allow for measurement. The remaining dual-cured core resin groups were in the order of CLD < CAD < CAS (*p* < 0.05). CAD showed a B/T ratio > 70% in the dual-cured group, and the CAS group showed a B/T ratio > 90%, whereas CLD showed a B/T ratio of approximately 40%.

### Vickers microhardness

The surface microhardness values at the top and bottom are listed in Table 3. Regarding the surface microhardness at the top of the specimen, the FTZ showed the highest hardness, and the BBB, which is a bulk-fill flowable resin composite, showed the lowest microhardness.

The microhardness values increased among the bulk-fill resins in the order of BBB, BMF, and BFO (*p* < 0.05). BFO showed a higher microhardness value than FMF in the control groups, and CLD, CAD, and CAS in the dual-cured core resin group; however, it showed a lower microhardness than FTZ.

For the dual-cured core resin composites, CLD exhibited a higher microhardness value than CAD, and CAD exhibited a higher microhardness value than CAS. It was confirmed that the microhardness of the top surface was higher with light-curing than without.

### Compressive strength

Table 4 presented the compressive strength of the experimental groups. CLS and BBB showed the lowest compressive strengths among the experimental groups, whereas BMF showed the highest compressive strength.

**Table 4:** **Compressive strength of the experimental groups (MPa)***n* = 12

Groups	Compressive strength (MPa)
FMF	239.16 (17.44) ^c^
FTZ	182.00 (43.68) ^b^
BBB	120.30 (8.08) ^a^
BMF	332.68 (33.27) ^d^
BFO	219.70 (16.38) ^c^
CLD	193.15 (35.35) ^b^
CLS	139.24 (25.50) ^a^
CAD	225.35 (25.91) ^c^
CAS	182.13 (27.74) ^b^

*n* = 12, Data are presented as mean (SD)

Mean values with different superscript lowercase letters represent statistically significant differences (*p* < 0.05)

The compressive strength of the bulk-fill resin groups followed the order: BBB < BFO and BMF (*p* < 0.05).

Among the dual-cured core resin groups, CLS exhibited the lowest compressive strength, followed by CAS, CLD, and CAD. CLD and CAD which were dual-cured groups of the dual-cured core resin composites, had significantly higher compressive strengths than the SC groups of the dual-cured core resin composites, CLS and CAS (*p* < 0.05).

## Discussion

To address the limitations of DOC, dual-cured resin composites have been introduced, aiming to combine the advantageous characteristics of SC and LC systems [6, 13, 14]. Additionally, due to time constraints and efficiency in placing core resin composite, bulk-fill resin composites have been introduced [18, 19]. These materials exhibit lower polymerization shrinkage and mechanical properties comparable to those of existing conventional resin composites [21, 22].

Contemporary bulkfill resins are available in two different visocosities on the dental material market. In this study, we investigated three bulkfill composite resins BBB, BMF and BFO. BBB and BMF are injectable flowable type of bulk-fill resin composites, and BFO is high-viscosity sculptable bulk-fill resin composite, which has nanofilled clusters as a filler composition. Thus, those two different viscosity characteristics were considered to require the corresponding different control resins, so we investigated two types of control composites: FMF and FTZ, which were flowable and nanofilled composite resins, respectively.

During the polymerization of resin composites, the intermolecular distances between dimethacrylate monomers, typically maintained by van der Waals attraction forces, decrease because of the conversion of C = C bonds and the formation of covalent C–C bonds [15]. Volumetric contraction was reported to last for approximately 24 h [1], but due to the limitations of the measuring machine, this study attempted to measure contraction for up to 1800 s to report the overall tendency (Fig. 2).

The trends observed during the polymerization process are shown in Figs. 1 and 2. In this study, the linear polymerization shrinkage of all resin composites initially increased rapidly upon light curing, reached a point of inflection after the completion of light exposure, and then gradually increased to reach a plateau (Fig. 1). This is because a portion of the polymerization shrinkage is offset by thermal expansion resulting from the heat generated by the polymerization reaction of the resin composite and light curing unit and only re-emerges after the completion of light exposure [13].

The SC groups (CLS and CAS) of the dual-cured resin composites had lower polymerization shrinkage rates than the dual-cured groups (CLD and CAD) (Fig. 3). This finding results from differences in the velocity of polymerization between the light-activation modes [1, 17]. Lee and Um [27] reported that when self-curing dual-cured resin, the curing speed was at least 15–322 times slower than when light-cured. Tauböck et al. [1] also reported that extending the pregel stage increased the time available for viscous flow, thereby alleviating polymerization shrinkage stress.

Additionally, in this experiment, the bulk-fill resin composites showed a lower polymerization shrinkage rate than the FMF control group (Fig. 3). The reduction in polymerization shrinkage of the bulk-fill resin composite can be attributed to the decrease in the proportion of the Bis-GMA monomer used or its replacement with Bis-EMA, TEGDMA, EBPDMA, and UDMA [28]. However, in comparison with the FTZ in the control group, the remaining bulk-fill resins, except for BFO, showed higher polymerization shrinkage (Fig. 3). Considering the previous suggestion that flowable resin composites generally shrink more than conventional resin composites [20], it seems reasonable to compare high- and low- viscosity resins.

Using microhardness evaluation, the B/T ratios were used to determine the DOC of the resin composites [11, 12, 14, 26, 28]. This is considered adequate when values equal to or higher than 80% are achieved [10, 26].

The study found that the B/T ratio of the bulk-fill resin composites, including BBB and BMF, reached 80%. The high-viscosity bulk-fill resin, BFO, also exhibited a value close to 80% (Table 3). This is as proof of the characteristics of the bulk-fill resin, which is designed to allow light to reach a depth of 4 mm, as claimed by the manufacturers.

The B/T ratio of CAS which is the SC group of core resin composites, showed the value of about 90%, but CAD, which is the dual-cured group of dual-cured core resin composite, fell short of 80% (Table 3). Surprisingly, in the case of CLD, which is a dual-cured group of core resin, a result of approximately 40% was obtained, and the CLS value was considered impossible to measure because the polymerization was insufficient after 24 h. (Table 3). The reason for this is believed to be the difference in the chemical compositions of the resins, listed in Table 1. Although not confirmed by each manufacturer, it could be speculated that the self-curing potential of Any-Core is greater than that of LuxaCore Z. Therefore, LuxaCore Z relies more heavily on light-polymerization, whereas Any-Core can achieve sufficient polymerization with less light-polymerization.

Mechanical properties are important because they resist mastication and parafunctional force [24–26]. In this study, the compressive strengths and microhardnesses of these materials were evaluated. Factors such as filler load, filler size and type, monomer composition, and network cross-linking density may affect the hardness of resin-based composites [14]. The conventional high-viscosity resin FTZ showed the highest hardness value on the top surface, while the low-viscosity bulk-fill resin, BBB, demonstrated the lowest hardness value of top surface. The properties of the resin are affected by the composition of the fillers and monomers (Table 1). Since the filler content of BBB was the lowest among bulk-fill resin composites, its microhardness was also the lowest, and as the filler content increased in the order of BMF and BFO, microhardness also increased.

The microhardness of the top surface of the dual-cured resin, CAD, had no difference with the CAS, although CAD was higher than that of CAS (*p* > 0.05). This is about Any-Core’s self-polymerization ability, which seemed to be the main mechanism in the polymerization process [14]. Conflicting results was appeared in the present experiment; while the top surface microhardness of the CLD could be measured, CLS measurements were not possible. The hardness values for the light-cured of dual-cured resins can vary depending on the material.

As the B/T ratio is determined by the hardness ratio of the upper and lower sides of each specimen, the microhardness values of the upper and the lower sides are crucial. In this study, the upper microhardness values of the CAS and CAD groups were not significantly different, whereas the lower microhardness values in the dual-cured group were significantly lower than those in the SC group (Table 3). Previous studies [1, 14, 17] have indicated that when dual-cured resins are exposed to light, certain monomers remain unreacted and become trapped within the partially gelled polymer network. This can inhibit the movement of free radicals, resulting in suboptimal polymerization of the lower part of the resin composite [13]. These findings clarify the observed phenomena.

Stress due to mastication exhibits compressive characteristics, and because core build-up materials require resistance to restoration, the compressive strength of the material is important for its mechanical properties [25]. All tested materials exceeded 50 MPa, and this compressive strength was clinically considered necessary for core formation [24, 29].

Compare the control group to bulk-fill resin, the compressive strength followed the order of BBB < FTZ < FMF ≈ BFO < BMF. Compressive strength is influenced by the filler content [24, 29]. In the case of bulk-fill resins, the decrease in filler content is aimed at enhancing the polymerization depth [10]. However, Table 1 shows that the bulk-fill resin composites used in this experiment had higher filler contents than FMF, which is a microfilled low-viscosity resin composite, and lower filler content than FTZ which is a nanofilled high-viscosity resin composite. The results of this experiment cannot be explained solely by the filler content.

This result was unexpected because differences in the filler content have been reported to affect the mechanical properties. However, mechanical properties are also affected by the filler size and type [24, 25, 29] One possibility for this finding could be related to the efficiency of photoactivation in providing adequate polymerization and cross-link formation that can compensate for the differences in mechanical properties between materials, which is expected owing to the differences in chemical composition.

The compressive strength of dual-cured resin varied depending on the material; however, the presence of light polymerization affected the compressive strength. Comparing the compressive strengths of dual-cured resins, CLS < CAS ≈ CLD < CAD were observed. Limited information about the initiator system of the materials used in this experiment prevents clear interpretation. However, dual-cured groups exhibit greater compressive strength than the SC groups, which appears to be due to the increased DC of the dual-cured groups.

One limitation of this study is that the total polymerization shrinkage measurement time was limited to 1800 s owing to the constraints of the measurement equipment. Previous studies have shown that polymerization shrinkage occurs over a 24-hour period, and the first hour of evaluation showed 89% of the total polymerization shrinkage [1]. Therefore, a longer measurement time of at least one hour would have been necessary to compare the polymerization shrinkage after polymerization was completed. The focus of this experiment was to identify the tendency rather than to evaluate the final value. Additional experiments that require extended observation periods are necessary.

In addition, the DC of the resin composite was indirectly evaluated using the B/T ratio, which is a commonly used method. The B/T ratio of the SC group was higher than the dual-cured group. When comparing the microhardness of the top surface and the compressive strength of the SC and dual-cured groups, the SC group exhibited poorer physical properties than the dual-cured group. Based on existing studies showing that the physical properties depend on the filler content and DC, the evaluation of DC through the B/T ratio could be a helpful tool for indirect evaluation of DC approximately. However, there are some limitations which were shown in this study. For direct and elaborate DC evaluation, the analysis by using spectroscopic methods such as Raman spectroscopy or Fourier-transform infrared spectroscopy is necessary in further study.

In conclusion, bulk-fill resins demonstrated significantly lower polymerization shrinkage compared to dual-cured core resin and conventional flowable resin. At a depth of 4 mm, bulk-fill resins consistently achieved an adequate B/T ratio. On the other hand, the DOC varied among different materials. Highly filled bulk-fill LC resins could be suggested as a reliable restorative material for deep cavities and post-endodontic core build-ups due to their polymerization shrinkage, DOC, and mechanical properties.

## Data Availability

Data is available from the corresponding author upon reasonable request.

## Abbreviations

LC: Light-Curing, Light-Cured
DOC: Depth Of Cure
DC: Degree of Conversion
SC: Self-Curing, Self-Cured
BFO: A group name for high-viscosity bulkfill resin; Filtek One Bulk fill (3M ESPE)
BBB: A group name for low-vistosity bulkfill resin; Bulk Fill Base (Sun Medical)
BMF: A group name for low-vistosity bulkfill resin; Metafil Bulkfill One (Sun Medical)
CLD: A group name for Core resin; Luxacore Z (DMG) with Dual-cure mode
CLS: A group name for Core resin; Luxacore Z (DMG) with Self-cure mode
CAD: A group name for Core resin; Any-Core (Mediclus) with Dual-cure mode
CAS: A group name for Core resin; Any-Core (Mediclus) with Self-cure mode
FTZ: A group name for conventional resin; Filtek Z350 (3M ESPE)
FMF: A group name for microfilled flowable resin; MetaFil Flo (Sun Medical)
LVDT: Linear Variable Differential Transformer
B/T ratio: Bottom/Top microhardness ratio
HV: microHardness Value
